# Looking on the bright side in social anxiety: the potential benefit of promoting positive mental imagery

**DOI:** 10.3389/fnhum.2014.00043

**Published:** 2014-02-04

**Authors:** Arnaud Pictet

**Affiliations:** Psychiatry, Experimental Psychopathology and Cognitive Therapies, University of OxfordGeneva, Switzerland

**Keywords:** social anxiety, mental imagery, cognitive bias modification, treatment, combined cognitive bias

## Abstract

Current cognitive models of social phobia converge on the view that negative imagery is a key factor in the development and maintenance of the disorder. Research to date has predominantly focussed on the detrimental impact of negative imagery on cognitive bias and anxiety symptoms, while the potential benefit of promoting positive imagery has been relatively unexplored. Emerging evidence suggests however that positive imagery could have multiple benefits such as improving positive affect, self-esteem and positive interpretation bias, and enhancing social performance. The present article defends the view that combining bias induction with a repeated practice in generating positive imagery in a cognitive bias modification procedure could represent a promising area for future research and clinical innovation in social anxiety disorder.

## Negative imagery in social anxiety

People with social anxiety disorder (SAD) frequently report experiencing negative self-imagery of how they think they appear to others in feared social situations (Hackmann et al., 2000). The assumption that this negative imagery may be important in maintaining social anxiety is a central tenet of most cognitive models of SAD (Clark and Wells, 1995; Rapee and Heimberg, 1997; Hofmann, 2007). For example, Clark and Wells (1995) model suggests that when socially anxious individuals enter a feared social situation, they shift their attention towards internal cues and use this information to infer how they appear to others. This internal information can often take the form of a distorted, negative self-image that individuals perceive as accurate.

Empirical support for the causal role of negative imagery in social anxiety has been established through a series of experimental studies (Hirsch et al., 2003, 2004; Stopa and Jenkins, 2007). Hirsch and colleagues conducted a series of experiments in which socially anxious participants were asked to hold an image in mind while taking part in two conversations with a stranger. During one conversation they were asked to hold in mind a negative image of the sort they would typically generate in anxiety provoking social situations, whereas during the other conversation they were instructed to hold a less negative (control) image in mind (based on a memory of a social situations in which they felt relaxed). Participants reported feeling more anxious when holding the negative image than the benign one. They also judged their social performance more negatively both in terms of showing more symptoms of anxiety and performing less well during the conversation.

Further support for the causal role of negative imagery in social anxiety has been provided in experimental studies conducted with low anxious participants. In Hirsch et al. (2005), participants who were normally confident public speakers were asked to generate a negative, positive or control (unrelated) self-image prior to giving a speech. Findings from socially anxious samples were replicated, with participants in the negative imagery condition reporting greater anxiety and judging their social performance as being poorer than those assigned to the positive imagery condition. A further line of inquiry explored how negative imagery influenced interpretation of ambiguous social situations. Examination of interpretative processes has particular relevance in social anxiety, as socially anxious individuals have been shown to exhibit a tendency to interpret ambiguous social events in a negative way (Clark and McManus, 2002). In Hirsch et al. (2003), participants were instructed to generate negative self-imagery while making “online” (i.e., as they read) interpretations of ambiguous social information. While participants assigned to a control (unrelated) task exhibited the usual non-threatening interpretation bias observed in non-anxious individuals (Hirsch and Mathews, 1997), those assigned to the negative self-imagery condition did not show such positive bias.

Interestingly, the link between imagery and bias was further shown to be reciprocal. In Hirsch et al. (2007), participants completed a repeated practice in accessing positive or negative interpretations of ambiguous social situations, a procedure known as “cognitive bias modification” (CBM; Mathews and Mackintosh, 2000). Following the completion of the CBM procedure participants were asked to imagine themselves in ambiguous social situations. Participants in the negative condition generated more negative self-imagery than those in the positive group, which demonstrated the reciprocal influence of bias on negative self-imagery. Additionally, when subsequently asked to imagine taking part in a stressful social situation, participants in the negative group rated their anticipated anxiety as being greater and their expected social performance as being poorer than those in the positive group. The bidirectional link shown between imagery and interpretation bias suggested the importance of examining the combined influence of cognitive biases in the maintenance of social phobia (Hirsch et al., 2006).

## Positive imagery in social anxiety

In line with the general emphasis in clinical research on negative cognitions and affect, the programme of research outlined above mostly focussed on delineating the influence of induced negative self-imagery on negative interpretation bias, ratings of social performance and social anxiety. The converse possibility that inducing positive imagery might lead to opposite and thus beneficial effects on social anxiety has been largely unexplored. There is accumulating evidence, however, that socially anxious individuals exhibit a range of positivity impairments, including reduced positive affect, absence of positive interpretation bias and deficits in approach-oriented behavior (Alden and Taylor, 2004; Kashdan, 2007; Weeks and Heimberg, 2012). Thus, targeting these positive deficits may represent an important target for future treatment of SAD.

Positive self-imagery represents a promising candidate to intervene on these positivity impairments in social anxiety. Mental imagery has been demonstrated to elicit stronger emotions than other forms of processing (Holmes and Mathews, 2010; Pictet and Holmes, 2013). This evocative power has further been shown to apply to both positive and negative emotions, leading to the suggestion that mental imagery may act as an “emotional amplifier” (Holmes et al., 2008a). Mental images have further been found to produce stronger emotions when they are generated from a field perspective, rather than from the perspective of an observer. This finding holds relevance in SAD given the tendency among socially anxious individuals to generate negative self-imagery from an observer perspective (Wells and Papageorgiou, 1999). Other properties of mental imagery with relevance to social anxiety have been identified. For example, imagining an event happening in the future has been shown to increase the perceived likelihood that the same event will actually occur in the future (Carroll, 1978). Further, generating positive imagery of the future has been shown to enhance motivation and goal-oriented behavior (Libby et al., 2007; Pictet et al., 2011). Taken together, these results suggest that promoting positive, field perspective imagery of the future in SAD may lead to improvements in anxiety symptoms, cognitive biases and motivation to engage in social behaviors.

Only a few studies to date have directly examined the impact of positive mental imagery in the context of social anxiety. In Stopa and Jenkins (2007) as well as Vassilopoulos (2005), participants who held a positive image in mind while giving a speech reported feeling less anxious and performed better than those who held a negative image. A limitation of this finding was that in the absence of a control condition, it was impossible to tell whether the effects found reflected the influence of positive imagery, negative imagery or a combination of the two. More recently, Hulme et al. (2012) explored the effects of experimentally inducing positive imagery or negative imagery on self-esteem, both during the imagery induction task and after performing a social threat task in which participants experienced social exclusion. Both high and low socially anxious participants who had held a positive image in mind reported higher levels of self-esteem at both times of the experiment than those who had held a negative image. A limitation of these findings was that the effects found were short-lived as confined to a single experimental session. Stopa et al. (2012) addressed this by testing whether an extended imagery practice involving seven daily sessions of positive imagery could lead to beneficial effects in socially anxious individuals. The imagery induction method used in the study was based on Hirsch et al. (2003) and consisted of the following two stages. Participants were first asked to generate a positive (or negative) image based on a social situation in which they had felt relaxed (or anxious). They were then asked to practice holding the positive (or negative) image in mind while listening to descriptions of ambiguous social situations (e.g., being called to reception in a Doctor’s surgery). Results indicated that after 1 week’s practice, participants in the positive condition reported higher levels of self-esteem and better performance ratings during a conversation with a stooge (in both subjective and objective assessments of performance).

The preliminary findings about positive imagery are encouraging but a replication on a clinical population is warranted. A common limitation in the research described was the absence of a control condition, which made the findings confusingly imputable to either negative or positive imagery, or a combination of the two. Further, little is known to date about the optimal way to promote positive imagery in SAD. The method employed in previous studies to induce positive imagery involved asking participants to recall a memory in which they had felt relaxed, then inviting them to close their eyes and ask them a series of questions designed to prompt imagery, such as how they thought they looked and sounded, how they felt and how they came across to other people. Whilst this method has been successful in producing the expected changes in previous research, there are many other possible ways to promote positive imagery and more research is needed to identify they key aspects of imagery than need to be harnessed. A potential caveat with regards to the methodology used was the questions used to prompt imagery (i.e., how they thought they look and came across to other people) may have favored the generation of observer perspective imagery. Encouraging the use of field perspective imagery (i.e., seeing the event through one’s own eyes) is likely to produce stronger effects on negative and positive emotions, and counteract the natural tendency among socially anxious individuals to imagine themselves from an observer perspective. A second caveat with regards to the methodology used is that it may be too challenging for individuals with clinical levels of social anxiety to recall and elaborate on a time when they had experienced a positive social situation. This possibility is supported by recent evidence showing that social anxiety is associated with an impairment in generating detailed imagery of past positive events (Moscovitch et al., 2011). One possible way to overcome this could be to train the generation of positive imagery in response to hypothetical, future-oriented scenarios rather than to past memories. Imagining the future involves similar psychological processes to remembering the past (Schacter et al., 2007), with the additional benefit that it may help reduce anticipatory anxiety and boost motivation to engage in future social activities.

## Positive imagery and cognitive bias modification (CBM) in social anxiety

The experimental research reviewed above evidenced a close and reciprocal relationship between imagery and negative interpretations of ambiguous social situations. In Hirsch et al. (2005), taking the perspective of a confident speaker rather than their own perspective was shown to block threatening inferences in people with high interview anxiety. However, it did not facilitate the generation of more benign interpretations. One possible explanation for this lack of effect on positive bias could be that participants may have struggled to adopt the perspective of a confident other as it was too far from what they would usually experience in similar situations. Another possible interpretation was that the induction procedure was too short-lived and thus not powerful enough to produce observable change in positive interpretation bias. By contrast, a more extensive practice of generating positive self-imagery in a CBM-I procedure could represent a more powerful way to produce changes in affect, cognition and behavior in a population of socially anxious individuals.

Only a few studies to date have explored the potential benefit of interpretation bias modification (CBM-I) for people with high levels of social anxiety (Murphy et al., 2007; Beard and Amir, 2008; Beard et al., 2011; Turner et al., 2011). Preliminary evidence supports the idea that CBM-I may be efficient at inducing a more benign (and less negative) interpretation bias and reducing anxiety symptoms in socially anxious individuals. However, very little is known about the durability of these effects as none of these studies included follow-up assessments. In the only attempt to combine CBM-I with mental imagery, Murphy et al. (2007) instructed high socially anxious participants to imagine themselves in a series of auditorily presented descriptions of social situations that started ambiguous but then were consistently resolved in a benign or negative way. Compared to a control condition where the same situations were presented but the outcome remained unspecified, participants who completed the benign interpretation training showed less negative and more positive interpretation biases, and further anticipated that they would be significantly less anxious in a future social situation. These effects were not imputable to group differences in state anxiety (as mood had been successfully equalized between the two groups following a filler task) and thus were directly due to the CBM-I procedure. Both the imagery and bias induction may have contributed to the positive effects found. At the time participants were presented with an upcoming social situation that could be potentially threatening (i.e., meeting two people they don’t know for a 5 min conversation), those who had previously completed the positive interpretation training may have been influenced by their newly acquired bias and interpret the situation in a more benign way (congruently with the training). Alternatively, participants may have spontaneously generated a positive image of them taking part in the upcoming social situation and thus have anticipated less anxiety.

The present article defends the view that combining a repeated practice in generating positive imagery with a CBM-I intervention may be a helpful component in future treatment of SAD. A similar hypothesis has been suggested in the context of depression (Holmes et al., 2009). Depression and social phobia are highly comorbid disorders (Ohavon and Schatzberg, 2010) and share common positivity impairments, such as reduced positive affect, lack of positive interpretation bias and deficit in positive imagery (Rude et al., 2002; Holmes et al., 2008b; Werner-Seidler and Moulds, 2011).

The effects of imagery-focussed CBM on depression have been tested using analog and clinical samples of depressed participants (Blackwell and Holmes, 2010; Pictet et al., 2011; Lang et al., 2012). In imagery-focussed CBM, participants first completed an introductory session in which they were trained to use imagery in a specific way (i.e., vivid, from a field perspective) and then were given a practice session of the CBM tasks on the computer. Throughout the training, participants were repeatedly prompted to focus on imagining the scenarios with as much vividness as possible. Pictet et al. (2011) found that within an analog sample of dysphoric participants, those who had completed a positive imagery-focussed CBM exhibited greater improvements in positive mood and greater performance on a behavioral task assessing approach motivation and persistence than those who had completed a control imagery condition. In the first clinical test of imagery-focussed CBM, Blackwell and Holmes (2010) administered a multi-session program involving seven sessions of CBM to a small sample of participants with a current major depressive episode. Imagery-focussed CBM was found to lead to significant improvements in depressive symptoms and cognitive biases, and these improvements were maintained at 2 weeks follow-up. Although promising, the findings were limited by the absence of a comparison condition, which left open the possibility that “non-specific” factors as well as spontaneous recovery might have intervened.

Lang et al. (2012) addressed this limitation by exploring the impact of a multi session imagery-focussed CBM-I compared to a closely matched control version of the program in a sample of currently depressed patients. Participants in the positive condition showed significantly greater improvement in depressive symptoms and cognitive bias from pre- to post-treatment compared to those in the control condition, and these improvements were maintained at 2 weeks follow-up. Of relevance for a potential application of imagery-focussed CBM to people with social anxiety, participants assigned to the positive imagery condition in Lang et al. (2012) reported significant reductions in trait-anxiety. More recently, Williams et al. (2013) tested the effects of an online version of imagery-focussed CBM in which seven daily sessions of CBM were followed by a 10-week internet-based cognitive-behavioral therapy (iCBT). Results showed that compared to participants assigned to a waitlist control, those assigned to the imagery-focussed CBM-I showed significant reductions in depressive symptoms, anxiety and distress. In addition, about a third of the participants showed clinically significant change after imagery-focussed CBM-I, while the proportion of clinical response reached 65% when combined with iCBT.

## Conclusion

SAD is a highly prevalent condition that causes considerable distress and significant functional impairments (Kessler et al., 2005). Effective psychological treatments exist but they are under-utilized due to the lack of qualified therapists and to the reluctance of some socially anxious people to disclose personal information to a stranger. This had led to increasing calls for the development of easily accessible and effective treatments (Clark et al., 2009). CBM procedures represent promising candidates as their format allows great flexibility and accessibility, and requires minimal clinical involvement. Early findings from clinical studies (Beard et al., 2011; Brosan et al., 2011; Amir and Taylor, 2012) suggest that CBM-I could be beneficial for socially anxious individuals, but more research is needed to determine the most efficient way to deliver CBM-I in clinical samples. Further, future research should include follow-up assessments to evaluate whether the effects of CBM are maintained over time. The research reviewed in this article converges on the proposition that using mental imagery could enhance the efficacy of CBM-I in SAD. One way in which we “resolve” the ambiguity of a social situation is by imagining the outcome. Hence, increasing access to positive and vivid representations of social situations in a CBM-I program could be a promising target for future treatments of SAD.

## Conflict of interest statement

The author declares that the research was conducted in the absence of any commercial or financial relationships that could be construed as a potential conflict of interest.
